# Cellular senescence induced by cholesterol accumulation is mediated by lysosomal ABCA1 in APOE4 and AD

**DOI:** 10.21203/rs.3.rs-4373201/v1

**Published:** 2024-05-14

**Authors:** Shaowei Wang, Boyang Li, Zhiheng Cai, Cristelle Hugo, Jie Li, Yi Sun, Lu Qian, Alan T. Remaley, Julia TCW, Helena C. Chui, David A. Bennett, Zoe Arvanitakis, Bilal Kerman, Hussein Yassine

**Affiliations:** University of Southern California; University of Southern California; University of Southern California; University of Southern California; University of Southern California; University of Southern California; BU: Boston University; University of Southern California; Boston University; University of Southern California; Rush University; Rush University; University of Southern California; University of Southern California

**Keywords:** Alzheimer’s disease, senescence, ABCA1, cholesterol, lysosome

## Abstract

**Background:**

Cellular senescence is a hallmark of aging and has been implicated in Alzheimer’s disease (AD) pathogenesis. Cholesterol accumulation drives cellular senescence; however, the underlying mechanisms are unclear. ATP-binding cassette transporter A1 (ABCA1) plays an important role in cholesterol homeostasis. ABCA1 expression and its trafficking is afiltered in APOE4 and AD cellular and mouse models. However, whether ABCA1 trafficking is involved in cellular senescence in APOE4 and AD remains unknown.

**Methods:**

We examined the association between cellular senescence and ABCA1 expression in human postmortem brain samples using transcriptomic, histological, and biochemical analyses. An unbiased proteomic screening was performed to identify targets that mediate cellular ABCA1 trafficking. APOE4-TR mice, immortalized, primary and induced pluripotent stem cell (iPSC) models were used to examine the cholesterol-ABCA1-senescence pathways.

**Results:**

Bulk and single nuclei transcriptomic profiling of the human dorsolateral prefrontal cortex from the Religious Order Study/Memory Aging Project (ROSMAP) revealed upregulation of cellular senescence transcriptome signatures in AD, which was strongly correlated with ABCA1 expression. Immunofluorescence and immunoblotting analyses confirmed increased ABCA1 expression in AD brain tissues, which was associated with lipofuscin-stained lipids and mTOR phosphorylation. Using discovery proteomics, caveolin-1, a sensor of cellular cholesterol accumulation, was identified to promote ABCA1 endolysosomal trafficking. Greater caveolin-1 expression was found in both APOE4-TR mouse models and AD human brains. Cholesterol induced mTORC1 activation was regulated by ABCA1 expression or its lysosomal trapping. Reducing cholesterol by cyclodextrin in APOE4-TR mice reduced ABCA1 lysosome trapping and increased ABCA1 recycling to efflux cholesterol to HDL particles, reducing mTORC1 activation and senescence-associated neuroinflammation. In human iPSC-derived astrocytes, the reduction of cholesterol by cyclodextrin attenuated inflammatory responses.

**Conclusions:**

Cholesterol accumulation in APOE4 and AD induced caveolin-1 expression, which traps ABCA1 in lysosomes to activate mTORC1 pathways and induce cellular senescence. This study provided novel insights into how cholesterol accumulation in APOE4 and AD accelerates senescence.

## Background

Cellular senescence, defined as permanent arrest of the proliferative state of the cell, is one of the hallmarks of aging and age-related diseases, including Alzheimer’s disease (AD) [1, 2]. Senescent cells secrete extensive inflammatory mediators, such as chemokines, cytokines, growth factors, and proteases, collectively known as the senescence-associated secretory phenotype (SASP) [3, 4], which contributes to AD pathology. Cellular senescence is triggered by many stimuli such as DNA damage, reactive oxygen species, telomere attrition, lysosomal dysfunction, tau and amyloid aggregation, and lipid accumulation [2, 5, 6]. Senescent astrocytes, microglia, endothelial cells, and neurons have been detected in the brains of patients with AD and AD mice [4, 7], particularly in relation to tau aggregation [8]. The clearance of senescent cells by senolytic therapy alleviates cognitive deficits in AD mouse models [9, 10]. In addition, removing senescent cells in naturally aged mice lengthens their lifespan [11]. However, the key drivers of cellular senescence and their association with AD neuropathology markers remain largely unknown.

Apolipoprotein E4 allele (APOE4) is the strongest genetic risk factor for late-onset AD [12, 13]. More senescent neurons were found in APOE4-target replacement (APOE4-TR) mice than in APOE3-TR mice [14]. Arguably, APOE4’s major effects in the brain are mediated by its cholesterol-transporting capability [15–17]. Cholesterol accumulation induces cellular senescence [18–20]. ATP-binding cassette A1 (ABCA1), a transmembrane protein, effluxes intracellular free cholesterol across the plasma membrane to lipidate ApoE and form HDL-like particles [21–23]. ABCA1 recycling to the plasma membrane is critical for cholesterol efflux into different apolipoproteins within brain cells [24]. We previously reported that APOE4 reduces ABCA1 plasma recycling and promotes its traficking to lysosomes in astrocytes [25]. A recent study found that lysosomal ABCA1 was necessary for inducing SASP in senescent fibroblasts. Rerouted ABCA1 imports cholesterol to lysosomes to form the mTORC1 scaffolding complex and induce SASP [5].

How cell-specific senescence signatures relate to brain ABCA1 expression, APOE4, and AD neuropathology remains unknown. To address this gap, we examined cellular senescence signatures using bulk-RNA and single-nucleus RNA sequencing (snRNA-seq) in postmortem human brain tissues from the Religious Order Study/Memory Aging Project (ROSMAP). This was followed by the measurement of senescence markers and ABCA1 expression in postmortem human brain tissues from individuals with or without AD, which differed by APOE genotype. Mechanisms of ABCA1 trafficking were investigated using cell lines, primary cells, human induced pluripotent stem cells (iPSC), and humanized APOE-TR mouse models.

## Methods

### Study design

To determine whether cellular senescence is associated with ABCA1 expression in APOE4 and AD, we analyzed the databases of bulk-RNA sequencing (632 participants) and single-nucleus RNA sequencing (427 participants) that performed from the dorsolateral prefrontal cortex (DLPFC) of postmortem human brain samples in the Religious Orders Study or the Rush Memory and Aging Project ROSMAP. Frozen mid frontal lobe brain tissues (138 participants) and formalin-fixed paraffin-embedded (FFPE) slides (12 participants) of the postmortem human brains from ROS were used to validate the findings of RNA-sequencing analysis. To identify the proteins that regulate ABCA1 degradation, proteomics was performed after ABCA1 purification in ABCA1 overexpression HeLa cells treated with recombinant ApoE3 or ApoE4 proteins. The biochemistry and histochemistry analysis were used to validate the findings from proteomics. To test the effect of cholesterol reduction on the APOE4-TR mice, 14–15 mice per group were determined by the publication. The mice were subjected to behavioral tests and treatment allocation was blinded to the testers. All animal experiments were approved by the Institutional Animal Care and Use Committee of the University of Southern California. Every effort was made to reduce animal stress and minimize animal use. For all experiments, the number of replicates, statistical tests used, and P values are reported in the figure legends.

### Analysis of bulk-RNA sequencing data

#### Database

Fifiltered raw counts were downloaded from the Synapse AD Knowledge Portal (https://www.synapse.org/#!Synapse:syn9702085) with Synapse ID: syn8456637.

Source data were collected from a cohort of 632 subjects from the Religious Orders Study or the Rush Memory and Aging Project (ROSMAP) [26]. Both studies were approved by the Institutional Review Board of Rush University Medical Center. All participants signed informed and repository consent forms and the Anatomical Gift Act. Based on the clinical diagnosis at the time of death, 83 study participants were diagnosed with no cognitive impairment (NCI), 162 with mild cognitive impairment (MCI), and 151 with AD dementia [27–29]. CERAD Braak staging represents the amount and distribution of neuritic plaques and neurofibrillary tangle pathology [30, 31]. The APOE genotype was determined as previously described [32]. Detailed information regarding the samples is provided in Supplementary Table 1.

#### Gene Set Enrichment Analysis (GSEA)

The R package GSVA (method option “ssGSEA”) was used to calculate the pathway enrichment score of each cell. The annotated gene sets were retrieved from MsigDB. REACTOME_CELLULAR_SENESCENCE (M27188) was selected to represent cellular senescence. The pathway score was tested to determine the differences between the variables of interest.

### Analysis of single nucleus-RNA sequencing data

#### Database

Post-QC counts and ROSMAP metadata were downloaded from the Synapse AD Knowledge Portal (https://www.synapse.org/#!Synapse:syn52293417) with Synapse ID: syn2580853. Source data were collected from a sample of 427 subjects from ROSMAP [33]. Detailed information about the samples is provided in Supplementary Table 2. Nuclei were isolated from frozen postmortem brain tissues and subjected to droplet-based single-nucleus RNA sequencing (snRNA-seq).

#### Gene Set Enrichment Analysis (GSEA)

The R package GSVA (method option “ssGSEA”) was used to calculate the pathway enrichment score of each cell. The annotated gene sets were retrieved from MsigDB. REACTOME_CELLULAR_SENESCENCE (M27188) and REACTOME_SENESCENCE_ASSOCIATED_SECRETORY_PHENOTYPE_SASP (M27187) were selected to represent cellular senescence and SASP. The linear mixed effect was used to test if the pathway score was different between the variables of interest, using the formula: ~ size factor (the library size of each cell) + interested variables + (projid (sample id)).

### SenTraGor (STG) staining

The formalin-fixed paraffin-embedded (PPFE) human brain slides containing middle frontal lobe region (ROS) were deparaffinized following the protocol (xylene, 3 min each time for two times; xylene 1:1 with 100% ethanol, 3 min each time for two times; 100% ethanol, 3 min each time for two times; 95% ethanol, 3 min each time for two times; 70% ethanol, 3 min each time for two times; 50% ethanol, 3 min each time for two times; running cold tap water to rinse for 5 min). The slides were then subjected to antigen retrieval using a Sodium Citrate buffer (10mM Sodium Citrate, 0.05% Tween 20, pH 6.0) for 30 min. The slides were washed once with TBS for 5 min and incubated with STG (Bio-Techne, #7555, 2mM, dissolved in 100% ethanol, filtered with 0.45uM filter) for 10 min at room temperature (RT). After incubation, the slides were washed twice with 50% ethanol followed by one time wash with TBST (0.025% Triton X-100 in TBS) for 5 min each wash. Then, primary antibodies (goat anti-biotin, anti-ABCA1, and anti-GFAP) were added to the slides and incubated overnight at 4°C. After washing three times with TBST, the slides were incubated with fluorescein-conjugated secondary antibodies at room temperature for 1 h. After washing three times with TBST, the slides were incubated with an autofluorescence quencher at room temperature for 5 min. After washing thrice with TBST, the slides were mounted with mounting medium (F4680; Sigma). Images were acquired using a slide scanner (Zeiss Axio Scan, Oberkochen, Germany). Z1, 20x objective) and analyzed using ImageJ software (NIH).

### Human brain tissue homogenate preparation

Frozen mid-frontal lobes of postmortem human brains were obtained from the Rush Alzheimer’s Disease Center (RADC) at the Rush University Medical Center. The Religious Orders Study (ROS) was approved by the Institutional Review Board (IRB) of Rush University Medical Center [34]. Detailed information on these samples is presented in Supplementary Table 3.

To extract soluble and insoluble proteins, frozen middle frontal lobe brain tissues were weighed and homogenized with TBS (1:15, w/v) containing a protease inhibitor cocktail and phosphatase inhibitor cocktail. The homogenate was centrifuged at 15,000 g for 1 h at 4°C. The supernatant was collected as the TBS fraction, and the pellets were incubated with TBSX (1% Triton X-100) (same volume as TBS) overnight with agitation at 4°C. The next day, the homogenate was centrifuged at 15, 000 × g for 1 h at 4°C and the supernatant was collected as the TBSX fraction. The protein concentrations of all fractions were measured using a bicinchoninic acid kit and used for further experiments. For ABCA1 detection, TBSX fraction samples were added to 4x Laemmli sample buffer (without boiling) and subjected to 12% SDS-PAGE for western blotting.

### Lysosome enrichment with LAMP-2 immunoprecipitation

LAMP-2 antibody (1.2μg) was added to the TBSX fraction samples (300uL) of AD E3/3 and AD E3/4 individuals. After incubation for 1 h at 4°C, 20μL Dynabeads Protein A/G (88802; Thermo Scientific) was added and incubated with rotation overnight at 4°C. After two washes with 1 mL of ice-cold 0.1% TBST, 20 μL of elution buffer (50mM Glycine, pH2.8) was added and incubated for 2 min at room temperature. The elution was quickly neutralized with 2μL of Tris buffer (1M, pH8.5). Then lamp-2 enriched samples were added to 4x Laemmli sample buffer (without boiling) and loaded onto a 7.5% SDS-PAGE gel for western blotting.

### ABCA1 complex purification

HeLa cells expressing ABCA1-GFP were cultured in DMEM (Corning, 10–013) with 10% fetal bovine serum (FBS) (Omega Scientific, FB-12) and 1% antibiotic-antimycotic (anti-anti) (Thermo Fisher, 15240062) at 37°C in a 5% CO2 incubator. When the cells were ready, recombinant APOE3 or APOE4 (0.2μM) was added for 4 h. After treatment, the cells were scratched, collected with DPBS (Corning, 21–031-CV), and washed twice with ice-cold DPBS. Then, 5 volumes of lysis buffer (0.5% NP40, 350mM Nacl, 20mM Hepes, 1.2% Triton X-100, complete cocktail) were added and incubated with rotation for 5 min at 4°C. The cell lysate was centrifuged for 15 min at 13,000 rpm and 4°C, and the supernatants were collected for immunoprecipitation.

100uL of GFP-Trap Agarose (gta-20, Chromotek) was first washed with IP buffer (PBS 7.5–7.9; 200mMNacl, 1mM EDTA, 0.5% Triton X-100, 1mM DTT and 0.2mM PMSF) three times and then added to 2mL supernatant of HeLa (ABCA1-GFP) cell lysates and incubated with rotation overnight at 4°C. The next day, agarose was washed with IP buffer three times with rotation at 4°C. After washing, a 2-fold volume of glycine (0.1M, pH 2.7) was used to elute the ABCA1 complex and neutralized with 1/10 volume of Tris-HCl (1M, pH 9.5) after elution. Parts of elution were used for SDS-PAGE analysis, and other parts were added with TCA (SA433–500, Fisher Scientific) to a final concentration of 20% and incubated for 15 min to precipitate proteins. After centrifugation (13,000rpm, 4°C, 30 min), the pellet was collected and washed once with 1mL of acetone (A949–1, Fisher Scientific). After removing the acetone, the protein pellets were airdry for 3–4 minute at room temperature to ensure that no liquid remained. Finally, the protein pellet was sent to a mass spectrometry facility (Harvard Center for Mass Spectrometry).

For ABCA1 degradation experiments, Hela cells expressing ABCA1-GFP were treated with recombinant APOE3 or APOE4 (0.2μM) (Academy Biomedical Company) for different time points. After treatment, cells were lysed with radioimmunoprecipitation assay (RIPA) buffer (9806, Cell Signaling Technology, CST), and total ABCA1 protein levels were detected by western blotting.

### Analysis of MS data

The normalized spectral abundance factors (NSAFs) [35] were used to measure the relative abundance and calculate the fold change for each protein. To obtain the NSAF value for a specific protein, the spectral abundance factor (SAF) value was first calculated by dividing the spectral count of the protein by its length (number of amino acids). The NSAF value was calculated by dividing the SAF value by the sum of the SAF values from all the identified proteins within each sample.

### Immunocytochemistry

Immortalized astrocytes were grown in 8-well cell culture chamber slides (Bioland Scientific, 07–2108). After fixing with 4% paraformaldehyde for 15 min, the cells were permeabilized with 1% Triton X-100 for 30 min and then blocked with 10% goat serum and 0.1% Triton X-100 for 1 h at room temperature. After blocking, the cells were incubated with anti-ABCA1 antibody (Abcam, ab18180) (1:100) and anti-Caveolin-1 antibody (CST, 3267S) (1:200) overnight at 4°C. After washing, the cells were incubated with Alexa Fluor Plus 488 labelled Goat anti-Mouse IgG (Thermo Fisher, A32723, 1:200) and Alexa Fluor 594 labelled Donkey anti-Rabbit IgG (Thermo Fisher, A21207) (1:200) for 1 h at room temperature in the dark. After washing, the nuclei were stained with Hoechst 33258 blue (Thermo Fisher, H3569) (1:12,000) for 5 min. After washing, the slides were mounted with mounting medium (F4680; Sigma). Images were captured using a Zeiss Axiovert 200M Inverted Fluorescence Microscope (20x objective). Images were analyzed using ImageJ software (NIH).

### Cell culture

Primary astrocytes were obtained from APOE3-TR and APOE4-TR mouse pups and cultured as previously described [60]. Briefly, cerebral cortices from 1 to 3 day-old neonatal mouse were dissected in ice-cold Hanks’ balanced salt solution (HBSS) (Corning, 21–021-CV) and digested with 0.25% trypsin for 20 min at 37°C. Trypsinization was stopped by adding a 2-fold volume of DMEM (Corning, 10–013) with 10% fetal bovine serum (FBS) (Omega Scientific, FB-12) and 1% antibiotic-antimycotic (anti-anti) (Thermo Fisher, 15240062). The cells were dispersed to a single-cell level by repeated pipetting and filtered through 100μm cell strainers (VWR, 10199–658). After filtering, the cells were centrifuged for 5 min at 1000 rpm and resuspended in culture medium (DMEM, Corning, 10–013) supplemented with 10% FBS and antibiotics. The cells were then seeded in a 75 cm^2^ flask and cultured at 37°C in 5% CO2. The medium was changed the next day and replaced every three days. The mixed glial cultures reached confluence after 7–10 days. The cells were then shaken at 250 rpm for 16h at 37°C to remove the microglia and oligodendrocyte progenitor cells. The remaining cells were harvested by trypsin digestion. At this stage, the culture contained 95% astrocytes, which were used for further experiments.

Immortalized mouse astrocytes derived from human APOE3 and APOE4 knock-in mice were gifts from Dr. David Holtzman and grown in DMEM/F12 (Corning, MT10090CV) containing 10% FBS, 1mM sodium pyruvate (Thermo Fisher, 11360070), 1mM geneticin (Thermo Fisher, 10131–035) and 1% anti-anti.

### Cell plasma membrane protein preparation

To study cell plasma membrane proteins, biotinylation of cell surface proteins was performed to isolated cell plasma membrane protein. Briefly, cells were washed twice with cold PBS followed by incubation with 0.5 mg/ml sulfo-NHS-SS-biotin (Thermo Fisher Scientific, PG82077) in PBS for 30 min at 4°C with shaking. The reaction was quenched by rinsing cells with 50 mM glycine in PBS. The cells were then lysed with RIPA buffer (CST, 9806) containing a protease inhibitor cocktail, followed by centrifugation at 12,000 × g for 10 min at 4°C. The supernatant was collected, and protein concentrations were measured using a BCA kit (Thermo Fisher, 23225). The radioimmunoprecipitation assay (RIPA) fraction represented the total protein fraction. Then, 100μg of the RIPA fraction from each sample was incubated with 40ul of NeutrAvidin agarose (Thermo Fisher Scientific, 29200) for 2 hours at 4°C. The agarose was then washed three times with PBS containing a protease inhibitor cocktail, followed by boiling with 100μL 2x sample buffer for 5 min at 95°C. After centrifugation (2,500 x g, 2 min, room temperature), the supernatants were collected as plasma membrane proteins. Membrane and total protein analyses were performed using western blotting.

### Immunoprecipitation

HeLa (negative) or HeLa (ABCA1-GFP) cells were lysed in RIPA buffer containing protease and phosphatase inhibitors. 10uL of GFP-Trap Agarose was added to 200uL cell lysates (1μg/μL) and incubated with rotation overnight at 4°C. The next day, agarose was washed with IP buffer three times with rotation at 4°C. After washing, a 2-fold volume of diluted (2x) sample buffer (Bio-Rad, 1610747) was added and boiled for 5 min at 95°C. After centrifugation (1,500 rpm, 5 min, room temperature), supernatants were collected for SDS-PAGE and western blotting.

Astrocytes were lysed in radioimmunoprecipitation assay (RIPA) buffer containing protease and phosphatase inhibitors. 1.5ug antibody (anti-ABCA1) was added to 400uL cell lysates (1μg/μL) and incubated with rotation for 1 h at 4°C. Then, 25uL Dynabeads Protein G (Thermo Scientific, 10004D) was added and incubated with rotation overnight at 4°C. After three washes with 1 mL of ice-cold 0.1% TBST, 25 μL of diluted (1.5 ×) sample buffer was added to the beads and boiled for 5 min at 95°C. The supernatants were collected and subjected to SDS-PAGE and western blotting.

The TBSX fraction of postmortem human cortical tissue was used to enrich the lysosomes. Anti-LAMP2 antibody was added to 300uL homogenate (1μg/μL) and incubated with rotation for 1 h at 4°C. Then, 20uL Dynabeads Protein A/G was added and incubated with rotation overnight at 4°C. After three washes with 1 mL of ice-cold 0.1% TBST, the beads were eluted with 20 μL elution buffer. The supernatants were collected and subjected to SDS-PAGE and western blotting.

### Western blotting

The cell lysates, brain homogenates, or immunoprecipitation protein complexes were separated on 4–15% mini-precast protein gels (Bio-Rad, 4561086) under reducing conditions and then transferred onto nitrocellulose membranes (Bio-Rad, 1704270). After transfer, the membranes were blocked with 5% fat-free milk (Bio-Rad, 1706404) in TBST for 1 h at room temperature, followed by overnight incubation with the primary antibody in 5% BSA at 4°C. The membranes were then incubated with an HRP-conjugated secondary antibody for 1 h at room temperature. A chemiluminescent HRP substrate (Millipore, WBKLS0500) was used for detection. The Fujifilm LAS-4000 imager system was used to capture images, and densitometric quantification was performed using Gel Quant NET software. The antibodies used are listed in Supplementary Table 5.

### siRNA and plasmid transfections

Non-targeted (NT), Caveolin-1 and AP2B1 siRNAs were obtained from Dharmacon. Cells were seeded in 60 mm dishes or 24-well plates and cultured overnight. The siRNAs (10–20nM) were transfected using the jetPRIME reagent (Polyplus Transfection, catalog #114). Protein expression was detected using western blotting 48 h after transfection.

The mouse Caveolin-1 (Myc-DDK-tagged) expression plasmid (MR201562) was purchased from Origene and purified using an endo-free plasmid DNA midi kit (Bioland Scientific, PD03–21). The pCMV-GFP expression plasmid was used as the control. The cells were seeded in 24-well plates and cultured overnight. The plasmid (0.125μg) was transfected using the jetPRIME reagent. Protein expression was detected using western blotting 48 h after transfection.

### Cholesterol efflux

Immortalized astrocytes were seeded in 24-well plate (0.12×10^5^ cells/500μL) and cultured overnight. The cells were transfected with 10nM siRNA for 24 h, followed by labeling with 1μCi/mL (3H) cholesterol (Moravek, MT9112) using serum-free DMEM/F12 containing 2 mg/ml fatty acid-free BSA (Sigma-Aldrich, catalog #A9647), and 2ug/ml acyl-coenzymeA: cholesterol acyltransferase inhibitor SANDOZ (Sigma-Aldrich, catalog #S9318) for 24 h. After washing once with serum-free culture medium, the cells were treated with recombinant CS-6253 peptide (1μM in DMEM/F12 containing 2 mg/mL fatty acid-free BSA, 2 μg/mL SANDOZ, and 200 μL/well) for 4 h. After treatment, the cell culture medium was collected and transferred to scintillation vials filled with 3 mL of the scintillation mixture. The cells were solubilized in 0.5 N NaOH (200 μL), neutralized with PBS, and then transferred to scintillation vials filled with 3 mL of scintillation mixture. After vigorous mixing, vials were counted using a Beckman LS6500 liquid scintillation counter (Beckman Coulter). The efflux of cholesterol was assessed by the ratio of cholesterol in the medium to the total cholesterol (medium and cell lysate).

### Cholesterol loading and depletion

Immortalized astrocytes or baby hamster kidney (BHK) cells were seeded in 24-well plates (0.5×10^5^ cells/500μL) and cultured overnight. Cells were washed twice with serum-free medium, and human LDL (10μg/mL diluted in serum-free medium containing 2 mg/mL fatty acid-free BSA) (Sigma, LP2–2MG) was added for 24 h. The cholesterol levels in the cells were determined using a cholesterol assay kit (filipin III staining, Abcam, ab133116). Cellular caveolin 1 protein levels were determined by western blotting.

Human iPSC astrocytes were seeded in 48-well plates (0.5×105 cell/300uL) and cultured for 48 h. Cells were first loaded with LDL as described above and treated with 1mM methyl-β-cyclodextrin (MβCD) for 2 h. Cellular cholesterol levels were determined using filipin staining. For parallel experiments, after MβCD treatment, cells were stimulated with TNFα (100ng/mL) and IFNγ (100ng/mL) for 16 h. RNA was extracted from each condition using qPCR.

### ABCA1 knock down cell line and cholesterol loading

ABCA1 knocking down astrocyte line was created by transfection of ABCA1 CRISPR Gene Knockout Kit (ABCA1-sgRNAs and Cas9 recombinant protein, Synthego). Three days after transfection, the single cell was seeded and cultured for two weeks. ABCA1 knocking out or knocking down clones were validated by western blotting using ABCA1 antibody.

ABCA1 WT or ABCA1 knockdown astrocytes were seeded in 24-well plates (0.5×105 cell/500uL) and cultured overnight. Cells were treated with methyl-β-cyclodextrin (MβCD, 5mg/mL) for 2 hours and then loaded with LDL (100μg/mL) for 2 hours. Then, the cells were lysed for further analysis.

### ABCA1 induction and cholesterol loading in BHK cells

BHK cells were seeded in 24-well plates (0.5×105 cell/500uL) and cultured overnight. ABCA1 expression was induced with Mifepristone (0.1nM) treatment for 16 hours. Then, cells with or without ABCA1 were loaded with LDL (100μg/mL) for 24 hours. The cells were lysed for further analysis.

### Cell lysate and mouse brain homogenate preparation

Immortalized or primary astrocytes were lysed with 1x RIPA buffer (CST, 9806) containing protease inhibitor cocktail (Sigma, P8340) and phosphatase inhibitor cocktail (Sigma, P0044), followed by centrifugation at 14,000 × g for 10 min at 4°C. The supernatant was then collected for further analysis.

The mouse cerebral cortex was weighed, and RIPA buffer containing a protease inhibitor cocktail and phosphatase inhibitor cocktail was added at a ratio of 1:30 (w/v). The tissue was then homogenized using a 2 mL glass Dounce tissue grinder, followed by centrifugation at 15, 000 × g for 1 h at 4°C. The supernatant was collected, and the concentration was measured using a BCA kit.

To extract soluble and insoluble proteins, mouse and human brain samples were weighed and homogenized with TBS (1:15, w/v) containing protease and phosphatase inhibitor cocktails. The homogenate was centrifuged at 15,000 g for 1 hour at 4°C. The supernatant was collected as the TBS fraction, and the pellets were incubated with TBSX (1% Triton X-100) (same volume as TBS) overnight with agitation. After centrifugation (15,000 × g, 1 hour at 4°C), the supernatant was collected as the TBSX fraction. The pellets were dissolved in 150–200uL guanidinium chloride (GnHCl) (5M, pH7.5) and incubated overnight at room temperature with agitation. After centrifugation (15,000 × g, 1 hour at 4°C), the supernatant was collected and dialyzed with a dialysis device (MINI Dialysis Device, 3.5 K MWCO, 0.1 mL, Thermo Scientific) in TBS overnight at 4°C. The solution was collected as the GnHCl fraction. The protein concentrations of all fractions were measured using a bicinchoninic acid kit and used for further experiments.

### Immunohistochemistry or immunofluorescence on mouse brain slides

Fixed mouse brains were sectioned using a Leica cryostat at a thickness of 10 μm. Slides were equilibrated for 30 min at room temperature and rinsed twice with PBS. After blocking with blocking buffer (10% goat serum + 1% BSA + 0.2% Triton X-100 in PBS) at room temperature for 1 hour, the sections were incubated with diluted primary antibodies (GFAP, Iba-1) and incubated overnight at 4°C. After washing, the slides were incubated in a 0.3% H_2_O_2_ solution in PBS at room temperature for 10 min to block endogenous peroxidase activity. Then, the SignalStain^®^ Boost IHC Detection Reagent (#8114, Cell Signaling Technology) was added to the slides and incubated at room temperature for 1 hour. After washing, DAB substrate solution was added to the slides and developed for 2–5 min until the desired color intensity was reached. After washing, the slides were mounted with mounting medium (F4680; Sigma). Images were taken using an Olympus Microscope (10x objective). Images were analyzed using the ImageJ software (NIH).

For immunofluorescence staining, after overnight incubation with the primary antibody (synaptophysin), the fluorescein labelled secondary antibody was added to the slides and incubated at room temperature for 1 hour (protected from light). After washing, the slides were mounted with mounting medium with DAPI (Sigma, F6057). Images were captured using a Zeiss Axiovert 200M Inverted Fluorescence Microscope (20x objective). Images were analyzed using the ImageJ software (NIH).

For ABCA1 and Lamp1 double staining, after overnight incubation with the primary antibody (ABCA1, 18180, Abcam; Lamp1, 99437, CST), the fluorescein labelled secondary antibody was added to the slides and incubated at room temperature for 1 hour (protected from light). After washing, the slides were mounted with mounting medium with DAPI (Sigma, F6057). Images were taken using a slide scanner (Zeiss, Axio Scan. Z1, 20x objective) and analyzed using the ImageJ software (NIH). Representative images were taken using Leica SP8 confocal microscope (60x objective).

### Lysosome isolation

Thirty to fifty mg of brain tissues were used to isolate lysosomes with the lysosome isolation kit (Minute Lysosome Isolation Kit, Invent Biotechnologies, LY-034) following the manuals. Briefly, the tissues were homogenate with a plastic rod for 1 minute, followed by the centrifugation at 16,000 X g for 30 seconds. The filter was discarded, and the pellet was resuspend by vigorously vertexing for 10 seconds, followed by centrifugation at 2,000X g for 3 minutes. The supernatant was collected to a fresh 1.5 ml microfuge tube and centrifuged at 4°C for 15 minutes at 9,000 X g. After centrifugation, the supernatant was carefully transfer to a fresh 1.5 ml tube and spined at 16,000 X g at 4°C for 30 minutes. After centrifugation, the supernatant was removed completely. Then, the pellet was resuspended in 200 μl of cold buffer A by pipetting up and down 60–100 times and vortex vigorously for 20 seconds, followed by centrifugation at 2,000 X g for 4 minutes. The supernatant was transferred to a fresh 1.5ml tube and added 100 μl of buffer B, and vortexed Briefly vortex to mix well (the supernatant to buffer B ratio is 2:1). The mixture was incubated on ice for 30 minutes and centrifuged at 11,000 X g for 10 minutes. After removing all the supernatant, the pellet (lysosomes) was resuspended in 50μl of RIPA buffer for further analysis.

### Bodipy-cholesterol stain

Fixed mouse brains were sectioned using a Leica cryostat at a thickness of 10 μm. Brain sections were incubated overnight at 4°C in a solution of 10 μg/ml bodipy-cholesterol (Cayman Chemical, 24618) in PBS. The slides were then washed with PBS 4 times. Images were obtained using a Zeiss Axiovert 200M Inverted Fluorescence Microscope (20x objective) using the same imaging parameters for each image. Images were analyzed using the ImageJ software (NIH).

### qPCR

The cells and brain specimens were harvested, and RNA was extracted using an RNA extraction kit (Thermo Fisher, K0731). cDNA synthesis was performed using the High-Capacity cDNA Reverse Transcription Kit (Thermo Fisher, 4368814). qPCR was performed using the PowerUp SYBR Green Master Mix (Thermo Fisher, A25742). The following primers were synthesized by Integrated DNA Technologies. Mouse Caveolin-1, TTCTCTTAAATCACAGCCCAGG (forward) and TGTAGATGTTGCCCTGTTCC (reverse); mouse beta-actin, ACCTTCTACAATGAGCTGCG (forward) and CTGGATGGCTACGTACATGG (reverse); mouse Nfkb-1, AAGACAAGGAGCAGGACATG (forward) and AGCAACATCTTCACATCCCC (reverse); mouse Nfkb-2, CACCCATCTAGTCACCAAGC (forward) and TCAGCACCAGCCTTTAGAAG (reverse).

mouse Rela, ACCCGAAACTCAACTTCTGTC (forward) and TTGATGGTGCTGAGGGATG (reverse); mouse Relb, GCTGTACTTGCTCTGTGACA (forward) and TGGCGTTTTGAACACAATGG (reverse); mouse Rel, ACCAGAACGCAGACCTTTG (forward) and TCGCAGTCTTCAATGTCCAG (reverse).

#### Isolation of cell types from mouse brain

Cell types isolation from mouse brain was performed following the published protocol [36]. Briefly, mice were euthanized with CO2 and perfused with PBS. The brains were harvest and cerebra were collected. Then, the cerebra were dissociated in the enzyme digestion mix (collagenase A (Sigma, 10103586001) + DNase I (Sigma, 10104159001) by passing three separate flame-polished Pasteur pipets to a single cell suspension. After centrifugation, the debris/myelin was removed by percoll (Sigma, GE17-0891-02) gradient centrifugation. The myelin fraction and total cells mixture were collected separately. Microglia from total cells mixture were purified by incubating with CD11b microbeads (Miltenyi Biotec,130-093-636) and passing LS magnetic column (Miltenyi Biotec, 130-042-401). CD11b negative population were also collected. After collection, the different cell types were lysate with RIPA for western blotting detection.

### Ion-mobility analysis

HDL particles in mouse CSF and plasma were determined by ion mobility analysis as previously reported [37]. Samples treated with dextran sulfate were introduced into a charge-reducing electrospray (TSI Inc., model 3482) every 13 min by automated loop injections using an integrated autosampler (Teledyne CETAC Technologies, model MVX-7100). The electrospray settings were as follows: voltage 2.0 kV, CO2 flow 0.15 slmp, and airflow 1.5 slmp. The differential mobility analyzer (TSI Inc., model 3085), coupled to a condensation particle counter (TSI Inc, model 3788), scanned particles 4.45 to 63.8 nm for 180 s. The generated data were analyzed using Fityk (version 1.3.1), as previously described, and graphed using OriginPro software (version 2021). Voigt probability distribution curves were generated from particle count (#/mL) vs. diameter range for lipoprotein subclasses and normalized by dividing subclasses by the sum of peak areas from all lipoproteins present within the spectrum.

### Animals and treatment

All animal experiments were approved by the Institutional Animal Care and Use Committee of the University of Southern California. Animals were housed under a standard 12-h light/dark cycle with water and chow diet in a pathogen-free animal facility at the University of Southern California. Every effort was made to reduce animal stress and minimize animal use.

ABCA1^fl/fl^ mice were obtained from Dr. John Parks of Wake Forest University School of Medicine. Nestincre mice (Strain #:003771) were purchased from Jax. ABCA1 conditional knockout mice were generated by crossing ABCA1^fl/fl^ mice with nestin-Cre mice. ABCA1 genotype was confirmed by PCR genotyping. Ten-month-old of wild type and ABCA1 conditional knockout mice were euthanized with CO_2_ and perfused with cold PBS. Brains were collected for further experiments.

The APOE3-TR and APOE4-TR mice were purchased from JAX. APOE3 and APOE4-TR mice of different ages were euthanized using CO_2_ and perfused with cold PBS. Brains were collected for further experiments.

APOE4-TR male and female mice (8 months old) were randomized to receive subcutaneous injections of 2 g/kg body weight 2-hydroxypropyl-β-cyclodextrin (HPCD) in phosphate-buffered saline (PBS) or PBS twice a week for eight weeks. The mice were then subjected to behavioral tests, and the testers were blinded to the treatment allocation. The injection was continued during behavioral tests (two weeks). Finally, after collection of the cerebrospinal fluid (CSF) and plasma, the mice were anesthetized and subjected to cardiac perfusion with ice-cold PBS. The brains were dissected, and one hemisphere was post-fixed in 4% paraformaldehyde (PFA) for cryostat sectioning, while the other was used for biochemistry assays and RNA purification.

### Novel Object Recognition (NOR)

This was performed as previously reported with some modifications [38]. On day 1, mice were acclimated to the test room for 1 h, followed by free exploration for 5 min in the test arena (without objects). On day 2, mice were placed into the same arena containing two identical objects and allowed to freely investigate both for 5 min (training trial). After an interval of two hours, the mice were placed back in the chamber with one novel object and one familiar object and allowed to explore for 5 min (testing trial). After each trial, the testing area and objects were thoroughly cleaned with 70% ethanol solution. The training and testing trials were recorded with a high-resolution camera, and the number of touches to both objects of each mouse in the testing trial was analyzed using the EthoVision XT software (Noldus). Mice touching the object with the nose were identified as one instance of nose touch. The movement distance, total touch times for both objects, and ratio of nose touch times of the novel object to the old object were calculated. One mouse in the HPCD group was excluded because of a fighting wound.

### Generation of human iPSC-astrocytes

APOE isogenic human iPSC–derived neural progenitor cells (NPC) were generated in the TCW laboratory [39]. Dissociated forebrain NPCs were differentiated into astrocytes in astrocyte medium (#1801, ScienCell), as previously described [40]. Briefly, forebrain NPCs were maintained at a high density on poly L-rrnithine hydrobromide (#P3655–50MG, Sigma) and laminin (#23017015, Thermo Fisher)-coated plates and cultured in NPC medium [DMEM/F12 (Corning, MT10090CV), 1xN2 supplement (Thermo Fisher, 17502048), 1xB27 supplement (Thermo Fisher, 12587010), 1 mg/mL laminin, and 20 ng/mL FGF2 (Thermo Fisher 13256029)]. The cells were split at approximately 1:3 to 1:4 every week with Accutase (Sigma, SCR005). NPCs were differentiated into astrocytes by seeding dissociated single cells at a density of 15, 000 cells/cm^2^ on Matrigel (Corning, 356255) -coated plates and cultured in complete astrocyte medium (#1801, ScienCell). After 30 days of differentiation, the astrocytes were ready for further experiments.

### Statistical analysis

GraphPad Prism software (version 10) or Program R (regression analysis, version 4.3.2) was used for all statistical analyses. One-way or two-way analysis of variance (ANOVA) was used to determine statistical significance, followed by Tukey’s test for multiple comparisons. All quantitative data are presented as mean ± SD. Statistical significance was defined as *p < 0.05, **p < 0.01, ***p < 0.001, ****p < 0.0001.

## Results

### ABCA1 expression associates with markers of cellular senescence in postmortem brain cells

To determine whether cellular senescence is associated with ABCA1 expression in APOE4 and AD, we first analyzed bulk-RNA sequencing data from the dorsolateral prefrontal cortex (DLPFC) of postmortem human brain samples from 632 participants in ROSMAP. The sample included 261 APOE3 controls (APOE3/3) and 96 APOE4 carriers (APOE3/4 and APOE4/4). Based on the clinical diagnosis using clinical data collected over time and proximate to the time of death, 83 participants were classified as having no cognitive impairment (NCI), 162 as having mild cognitive impairment (MCI), and 151 as having AD dementia. A detailed neuropathological evaluation included Braak staging, which indicated the degree of AD pathology based on the severity of tau tangles. A total of 164 participants were classified as Braak Stages 0, I, II, and III. A total of 116 patients were classified as Braak stage IV disease. A total of 116 participants were classified into Braak stages V and VI. The CERAD score, which is a semiquantitative measure of neuritic plaques, was used to classify patients as neuritic plaque positive (*n = 241;* scores 1 and 2) and negative (*n = 155*; scores 3 and 4), as previously published [31]. The additional clinical and pathological characteristics of the samples are presented in Supplementary Table 1.

**Figure 1:**
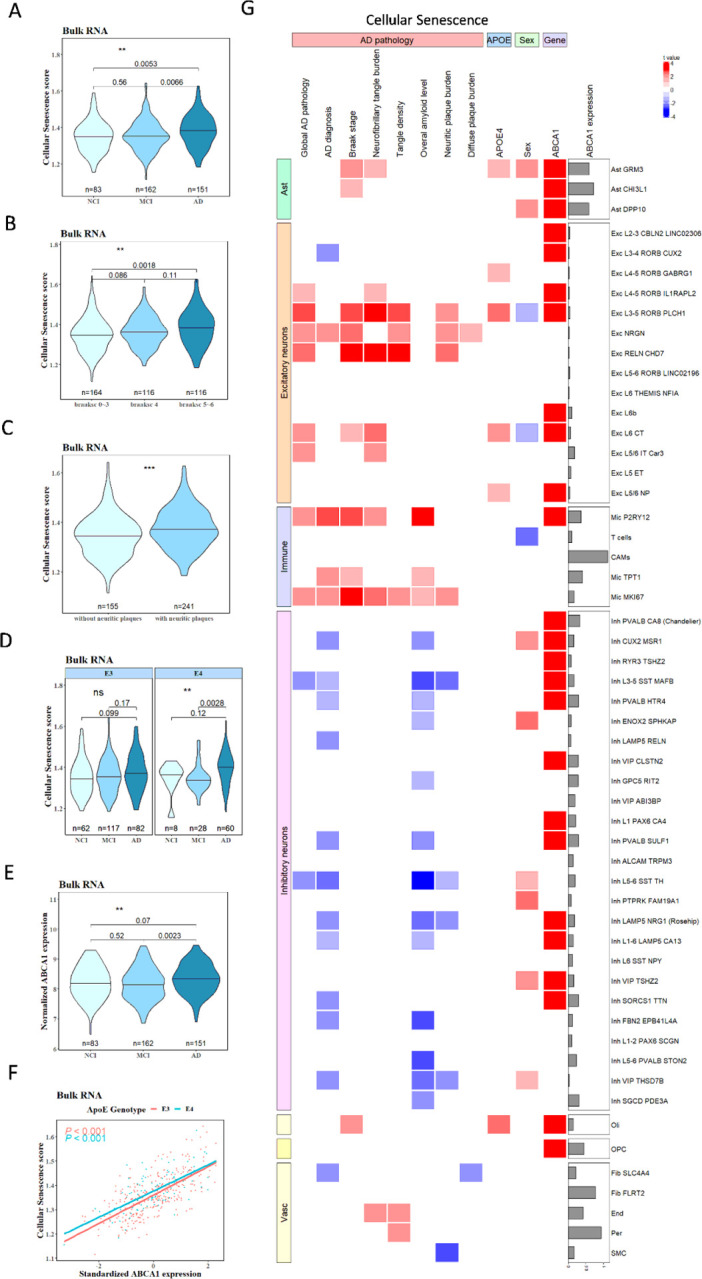
Bulk-RNA and single-nucleus RNA (sn-RNA) sequencing analyses revealed an association between ABCA1 and cellular senescence in APOE4 and AD. (**A**) Bulk-RNA sequencing analysis of cellular senescence ssGSEA scores in the three clinical groups. (**B**) Bulk-RNA sequencing analysis of cellular senescence ssGSEA scores in the different Braak stage groups. (**C**) Bulk-RNA sequencing analysis of cellular senescence ssGSEA scores between the groups with and without neuritic plaques. (**D**) Bulk-RNA sequencing analysis of cellular senescence ssGSEA scores by different APOE genotypes. (**E**) Bulk-RNA sequencing analysis of ABCA1 expression in the three clinical groups. (**F**) Association between ABCA1 expression and cellular senescence ssGSEA score in the bulk-RNA sequencing analysis using a linear model. (**G**) Single-nucleus RNA sequencing analysis of the association of cellular senescence ssGSEA scores in different brain cells with factors, including AD pathology, sex, APOE genotype, and ABCA1 expression. The association in the heatmap is represented by the t value (the effect size **β** divided by standard error of **β**). In the heatmap, red indicates significant positive associations, blue indicates significant negative associations, gray indicates non-significant associations, and cutoff p=0.1). The gray bar plot represents ABCA1 expression levels in different cell types. Data were analyzed using an unpaired t-test (**C**) or one-way ANOVA (**A, B, D, E**) and alinear model (**F**). **p<0.01, ***p<0.01.

The cellular senescence pathway enrichment score for each sample was calculated using single-sample Gene Set Enrichment Analysis (ssGSEA). Each pathway enrichment score represents the relative expression level of all genes in a specific pathway within the sample. The cellular senescence pathway enrichment scores were analyzed to determine whether there were differences between the participant parameters of interest. Cellular senescence ssGSEA was higher in the AD dementia group than in the NCI or MCI group (*p = 0.0053*, AD vs. NCI; *p = 0.0066*, AD vs. MCI; Fig. 1A). In addition, cellular senescence ssGSEA increased with advanced Braak stages (Fig.1B) and CERAD (Fig.1C). The greater cellular senescence score was driven by APOE4 carriers with AD dementia compared with APOE4 carriers without AD dementia, whereas no difference was found among APOE3 carriers (Fig.1D). Similar to the senescence ssGSEA scores, ABCA1 mRNA levels were higher in the AD dementia group than in the NCI or MCI groups (Fig.1E). Cellular senescence ssGSEA was positively correlated with ABCA1 expression independent of the APOE genotype (*p < 0.001*, Fig.1F).

To obtain cellular level information, we further analyzed single-nucleus (sn) RNA sequencing data from 2.3 million nuclei of 427 individuals from ROSMAP[33] in relation to AD neuropathology scores and ABCA1 expression. The cohort included patients without AD dementia (*n = 189*) and AD dementia (*n = 238*). Based on the APOE genotype, the participants were classified as APOE4 non-carriers (APOE2/2, APOE2/3, and APOE3/3) and APOE4 carriers (APOE2/4, APOE3/4, and APOE4/4). We tested whether cellular senescence is associated with AD characteristics (Global AD pathology: a quantitative summary of AD pathology derived from counts of three AD pathologies: neuritic plaques, diffuse plaques, and neurofibrillary tangles; Braak stage: a semiquantitative measure of distribution and severity of neurofibrillary tangle pathology; tangle density: immunohistochemistry for phosphorylated tau; overall amyloid level: immunohistochemistry for amyloid beta) and other AD risk factors, such as APOE4, sex, and ABCA1 mRNA expression. The association between the cellular senescence score and the variables of interest was examined using a linear mixed-effect model: size factor + variable + sample ID for each by sub-cell type. The T-values are presented in a heat map (Fig. 1G) represent the effect size **β** devided by standard error of **β** The sample characteristics are listed in Supplementary Table 2.

AD pathological factors, such as neurofibrillary tangle burden and neuritic plaque burden, were associated with increased cellular senescence ssGSEA scores in astrocytes, microglia, excitatory neurons, oligodendrocytes, and vascular cells (Fig. 1G). Sex was also associated with cellular senescence ssGSEA scores in astrocytes and inhibitory neurons (Fig. 1G). APOE4 was associated with cellular senescence ssGSEA scores in GRM3 (glutamate metabotropic receptor 3 positive) astrocytes, some excitatory neuron types, and oligodendrocytes (Fig.1G). Importantly, ABCA1 expression was strongly associated with cellular senescence ssGSEA scores in most brain cell types including astrocytes, excitatory neurons, microglia, inhibitory neurons, oligodendrocytes, and oligodendrocyte progenitor cells (Fig. 1G). Similar pro les showed that ABCA1 expression was correlated with SASP ssGSEA scores in the same postmortem human brain samples (Supplementary Fig. 1A). Sn-RNA sequencing analysis also indicated that mTOR ssGSEA scores were positively associated with ABCA1 expression in astrocytes, excitatory and inhibitory neurons, microglia, oligodendrocytes, and oligodendrocyte progenitor cells (Supplementary Fig. 1B). AD characteristics such as Braak stage, NFT burden, and neuritic burden were also positively correlated with mTOR ssGSEA scores in excitatory neurons (Supplementary Fig. 1B). APOE4 was associated with increased mTOR expression in excitatory neurons, oligodendrocytes, and pericytes (Supplementary Fig. 1B). Taken together, these results indicate that in participants with AD dementia, particularly those with APOE4, cellular senescence scores were strongly correlated with ABCA1 expression and mTOR activation across different cell types.

#### Markers of cellular senescence and lysosomal ABCA1 proteins are increased in APOE4 AD human brain tissues

**Figure 2:**
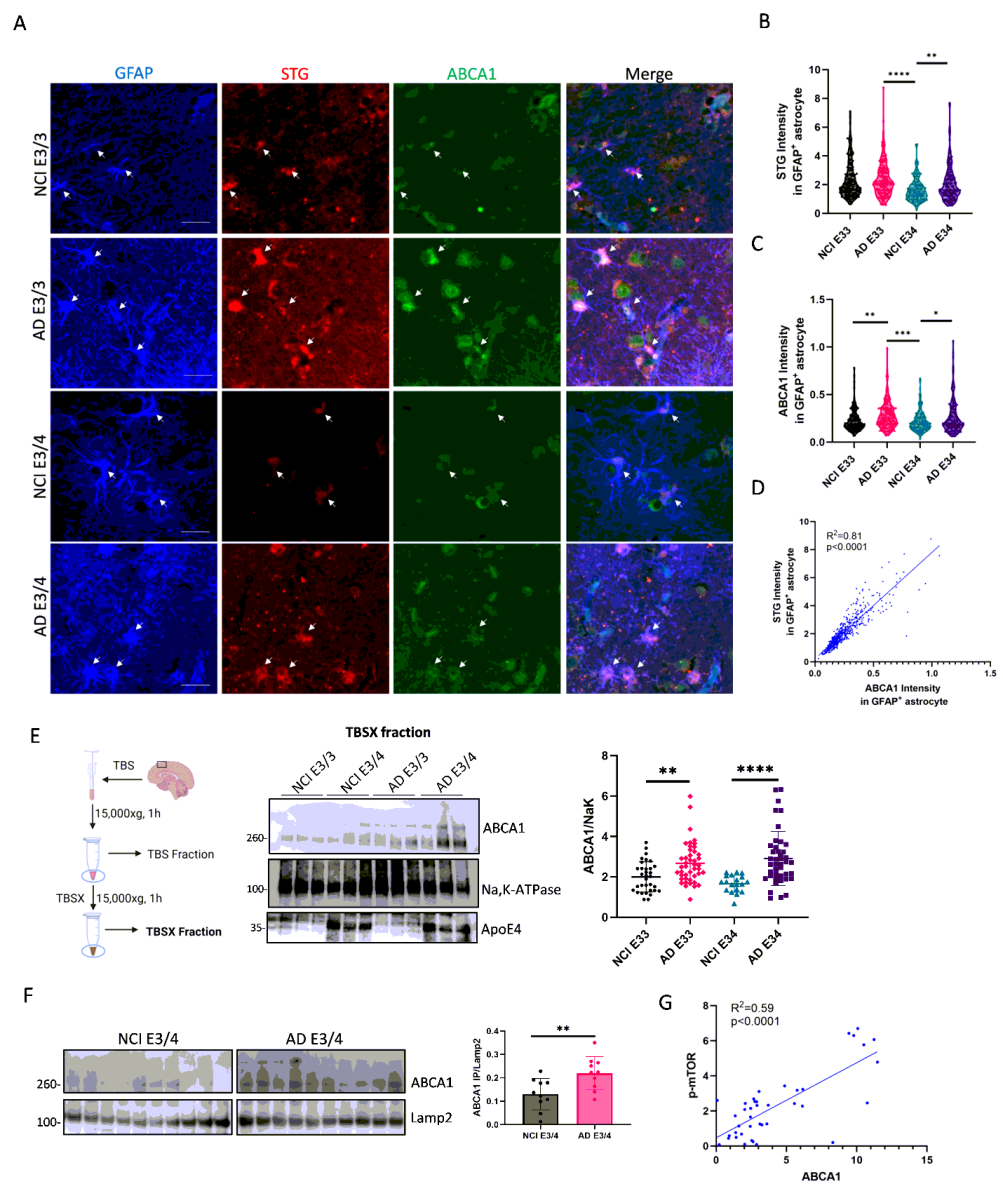
ABCA1 protein levels are associated with senescence markers in post-mortem AD human brains. All postmortem human brain tissues and slides were obtained from mid-frontal lobe tissue sections from the ROS. (**A**) Images of GFAP, STG, and ABCA1 staining in representative slides, with or without AD and APOE4. Arrows indicate GFAP-positive astrocytes that were also positive for ABCA1 and STG. (**B, C**) Quantification of STG intensity (**B**) and ABCA1 intensity (**C**) in GFAP-positive astrocytes on slides (n=3 individuals, n=138–160 astrocytes; A.U.). (**D**) Association between ABCA1 and STG intensity in GFAP-positive astrocytes (n=12, n=590 astrocytes). (**E**) Total membrane protein from human brain tissue was extracted using TBSX buffer (TBS with Triton X-100). ABCA1 protein levels in the TBSX fraction were detected by WB. Quantification of total membrane ABCA1 protein levels (NCI APOE3/3, n=33; NCI APOE3/4, n=19; AD APOE3/3, n=44; AD APOE3/4, n=42). (**F**) ABCA1 and Lamp2 were measured by WB after Lamp2 was immunoprecipitated from the homogenate of human brain tissues (n=10 per group). (**G**) Phosphorylated mTOR and ABCA1 protein levels in human brain tissue homogenates were measured by WB (Fig.S2D). The association between phosphorylated mTOR and ABCA1 protein levels was analyzed using simple linear regression (n=40). Data are represented as mean± SD and were analyzed using two-tailed t-test or one-way ANOVA followed by Tukey’s test. *p<0.05, **p<0.01, ***p<0.001, ****p<0.0001.

Lipofuscin accumulation is a marker of non-degradable lipid and protein aggregates in the cytoplasm of stressed or damaged cells and is one hallmark of cellular senescence [41]. To confirm our RNA-seq findings, we stained postmortem human brain slices (mid-frontal lobe region) from the Religious Order Study (ROS) with SenTraGor (STG, biotinylated Sudan black that detects lipofuscin) [42], anti-ABCA1 antibody, and anti-GFAP antibody (astrocyte marker) (Fig.2A). Validation of STG and ABCA1 staining is shown in Supplementary Fig. 2A. The STG signal in GFAP-positive astrocytes was stronger in APOE3/3 and APOE3/4 carriers with AD dementia compared to APOE3/4 carriers with NCI (Fig. 2B). No difference in the STG signal was found between NCI APOE3/3 and AD APOE3/3 or between APOE3/3 and APOE3/4 in either the NCI or AD dementia groups (Fig. 2B). ABCA1 expression in GFAP astrocytes was higher in the APOE3/3 and APOE3/4 AD dementia groups compared to APOE3/3 and APOE3/4 NCI groups (Fig. 2C). No difference in ABCA1 expression was found between the APOE3/3 and APOE3/4 genotypes in either the NCI or AD group (Fig. 2C). Importantly, the STG signal in astrocytes strongly correlated with ABCA1 expression (Fig. 2D, R^2^ = 0.81, *p < 0.0001*), confirming the association between ABCA1 expression and cellular senescence.

Next, ABCA1 protein levels were examined in proteins extracted from postmortem human middle frontal lobe tissues of individuals with NCI and AD dementia that differed by APOE4 status and who were enrolled in ROS. The tissue characteristics of the samples are listed in Supplementary Table 3. Because ABCA1 is predominantly localized in cell membranes [43], brain tissues were homogenized in TBS to remove cytosolic proteins, and proteins were extracted using 1% Triton X-100 in TBS (TBSX) (Fig. 2E). The TBSX fraction contained all membrane proteins, including plasma and organelle membranes. As expected, ABCA1 was found to be enriched in the TBSX fraction (Supplementary Fig. 2B). Total membrane ABCA1 levels were higher in APOE3/3 and APOE3/4 carriers with AD dementia than in APOE3/3 and APOE3/4 carriers in the NCI group (Fig. 2E). However, no differences in ABCA1 levels were detected between the APOE3/3 and APOE3/4 genotypes in either the NCI or AD groups (Fig.2E, full blots are shown in Supplementary Fig.2 C). Immunoprecipitation for LAMP-2 (a membrane marker of lysosomes) revealed that more ABCA1 was bound to LAMP-2 in the AD APOE3/4 group than in the NCI APOE3/4 group, indicating that more ABCA1 was trapped in the lysosomes in APOE4 AD (Fig.2F).

To validate the association between ABCA1 and mTOR activation, we measured phosphorylated mTOR and ABCA1 protein levels in postmortem human brain ROS samples (Supplementary Fig. 2D). Phosphorylated mTOR levels positively correlated with ABCA1 levels (Fig. 2G; R^2^ = 0.59; *p < 0.0001*), confirming the association between ABCA1 and mTOR observed in transcriptomic data.

### Proteomics identify caveolin-1 as a mechanism for ABCA1 trafficking to lysosomes following ApoE4 treatment

**Figure 3:**
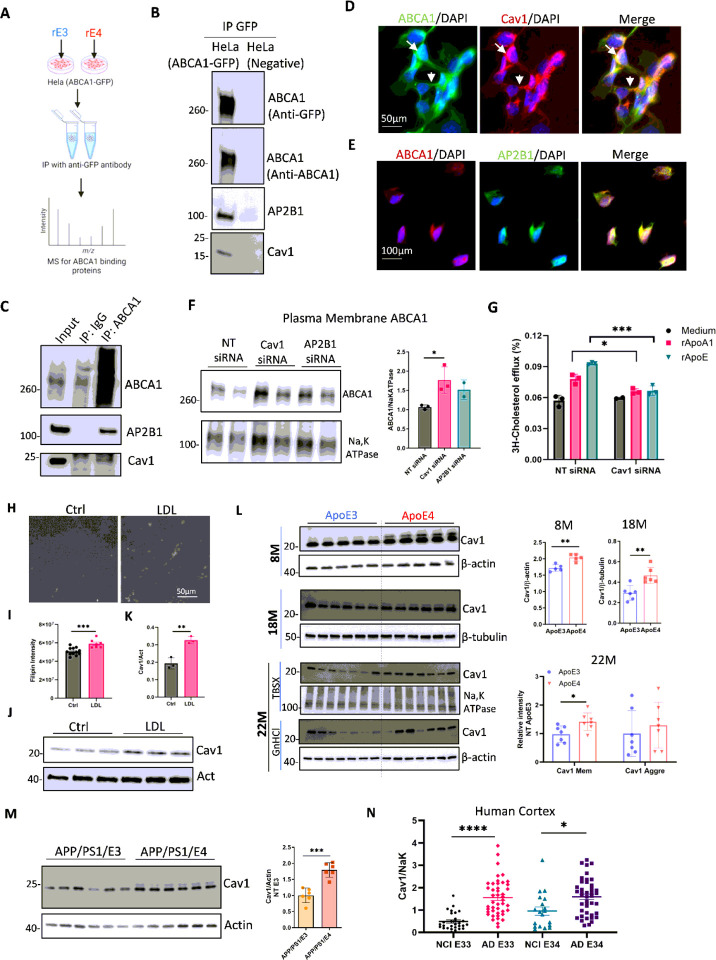
Caveolin-1 regulates ABCA1 trafficking, and its expression is increased in APOE4 and AD cells. **(A)** Schematic overview of the workflow for purification of the ABCA1 complex. (**B**) Co-immunoprecipitation of ABCA1 in ABCA1-GFP expressing and wild-type HeLa cells. (**C**) Co-immunoprecipitation was performed in the lysates of immortalized astrocytes using an ABCA1 antibody or species-matched IgG. ABCA1, Caveolin-1 and AP2B1 were detected by immunoprecipitation. (**D**) Co-staining for ABCA1 and caveolin-1 in immortalized astrocytes. Arrflow indicates the cytoplasm, and arrowhead indicates the plasma membrane. (**E**) Co-staining for ABCA1 and AP2B1 in immortalized astrocytes. (**F**) Mouse primary astrocytes were transfected with non-target (NT), caveolin-1, and AP2B1 siRNA for 48 h. Plasma membrane protein was enriched by biotin agarose beads and the ABCA1 protein levels were detected by WB. Quantification was performed in two–three independent experiments. (**G**) Immortalized astrocytes were transfected with non-target or caveolin-1 siRNA for 24 hours followed by labeling with 3H-cholesterol for 18 hours. Cholesterol efflux was measured after treatment with recombinant ApoA1 or ApoE for 4 hours (n=3 biological replicates). (**H**) Filipin staining of immortalized astrocytes loaded with or without low-density lipoprotein (LDL) (10μg/mL) for 24 h. (**I**) Quantification of filipin intensity in (**H**) (n=7–12 random areas from two cultured wells). (**J**) Caveolin-1 protein levels in immortalized astrocytes loaded with or without LDL (10μg/mL) for 24 h were detected by WB. (**K**) Quantification of caveolin-1 expression (**J**). (n=3 biological replicates). (**L**) Protein levels of caveolin-1 in the cortex of 8-months-old and 18-months-old APOE3 and APOE4-TR mice (n=5–6 mice for each genotype, mixed gender). For 22-months-old mice, the cortex was homogenized with TBS and TBS containing 1% Triton X-100 (TBSX) and then dissolved with guanidinium chloride (GnHCl). Caveolin-1 protein levels in the soluble membrane protein-enriched fraction (TBSX) and aggregated protein-enriched fraction (GnHCl) were detected by WB (n=7 mice for each genotype, both sexes). (**M**) Protein levels of caveolin-1 in the cortex of 6-months-old APP/PS1/APOE3 and APP/PS1/APOE4 mice (n=6 mice of each genotype, both sexes). (**N**) Total membrane caveolin-1 protein levels in human postmortem mid-frontal lobe tissues from reactive oxygen species (ROS) were detected by WB. Quantification of the relative intensity of caveolin-1 normalized to the plasma membrane marker Na, K-ATPase (NCI APOE3/3, n=29; AD APOE3/3, n=44; NCI APOE3/4, n=19; AD APOE3/4, n=42). Data are represented as mean ±SD and were analyzed using two-tailed t-test or one-way ANOVA followed by Tukey’s test. *p<0.05, **p<0.01, ***p<0.001, ****p<0.0001.

Our findings indicated that APOE4 trapped ABCA1 in lysosomes in the human brain with APOE4 and AD dementia. We previously reported that APOE4 traps more ABCA1 in the lysosomes of astrocytes than does APOE3 [25]. To explore the underlying mechanisms, we treated ABCA1-GFP overexpressing HeLa cells with recombinant ApoE3 (rE3) or ApoE4 (rE4). rE4 treatment resulted in lower total ABCA1 protein levels than rE3 treatment starting at 3 h, indicating greater ABCA1 degradation with rE4 (Supplementary Fig. 3A). The cells were then treated with either rE3 or rE4 for 4 h. Cell lysates were incubated with GFP-agarose to enrich ABCA1-binding complexes for proteomic analysis using liquid chromatography–mass spectrometry (LC-MS/MS) (Fig. 3A). SDS-PAGE with silver staining confirmed the successful enrichment of ABCA1 complexes. A similar size distribution profile of the ABCA1 complexes was observed between rE3 and rE4 treatments (Supplementary Fig. 3B). MS analysis revealed that 768 proteins among the ABCA1 co-precipitated complexes were shared by rE3 and rE4 treatments, 347 proteins were unique to the rE3 treatment, and 314 proteins were unique to the rE4 treatment (Supplementary Fig. 3C). We performed Kyoto Encyclopedia of Genes and Genomes (KEGG) pathway analysis of these 768 shared proteins using the Database for Annotation, Visualization, and Integrated Discovery (DAVID) platform [44] to identify specific pathways mediating ABCA1 degradation. The top 14 pathways identified based on the number of enriched proteins and p-values are shown in Supplementary Fig. 3C. Subsequent experiments focused on the endocytic pathway, given its potential role in protein degradation [45, 46]. 27 proteins were enriched in the endocytosis pathway, and nine of these proteins (Supplementary Table4) showed greater expression in the rE4 treatment group than in the rE3 group (Supplementary Fig. 3D). Internalization of surface ABCA1 has been reported to be regulated by multiple endocytosis mechanisms, including clathrin-mediated, caveolin-mediated, and GTPase ADP ribosylation factor 6 (ARF6) mediated pathways [47]. We selected caveolin-1 (Cav-1), linked to caveolae-dependent endocytosis, and the adaptor-related protein complex 2 subunit beta 1 (AP2B1), linked to clathrin-dependent endocytosis [45] for further validation by co-immunoprecipitation experiments. Caveolin-1 and AP2B1 were detected in the elution complex of ABCA1-GFP expressing HeLa cell lysates but not in control HeLa cell lysates (Fig. 3B).

Co-IP in astrocyte lysates revealed that caveolin-1 and AP2B1 were pulled down by the ABCA1 antibody, but not by control IgG (Fig. 3C). ABCA1, caveolin-1, and AP2B1 colocalized in both the membrane and intracellular compartments of astrocytes (Fig. 3D, and 3E, respectively). To test the effects of AP2B1 and caveolin-1 on ABCA1 degradation, caveolin-1 and AP2B1 expression were reduced by siRNA in mouse primary astrocytes. Forty-eight hours after transfection with siRNA, the cells were labelled with biotin and the membrane proteins were purified with avidin agarose beads. Caveolin-1 and AP2B1 siRNAs reduced the expression of the target proteins (Supplementary Fig. 3E). Reducing caveolin-1, but not AP2B1, increased plasma membrane ABCA1 levels in mouse primary astrocytes (Fig. 3F), supporting that caveolae-mediated endocytosis regulated ABCA1 degradation. To test the effect of caveolin-1 on ABCA1-induced cholesterol efflux, we labeled astrocytes transfected with caveolin-1 siRNA and 3H-cholesterol and used recombinant ApoA1 or ApoE3 as cholesterol acceptors. Surprisingly, the results showed that reducing caveolin-1 expression significantly decreased ABCA1-mediated cholesterol efflux (Fig. 3G) despite the increase in total ABCA1 protein levels after reducing caveolin-1 expression (Supplementary Fig. 3F). Indeed, overexpression of caveolin-1 in astrocytes increased the cholesterol efflux (Supplementary Fig. 3G). These results indicate that caveolin-1 not only traffics ABCA1 to the lysosome, but also plays a critical role in promoting ABCA1 mediated cholesterol efflux possibly by enriching the plasma membrane with cholesterol or participating in the bidirectional recycling of ABCA1 between the membrane and intracellular pools.

### The association of APOE4 with increased caveolin-1 expression is a marker of cholesterol accumulation

Caveolae are vesicular invaginations of the plasma membrane that increase with cellular cholesterol accumulation [48]. We tested whether cholesterol loading in two independent cell lines modulated caveolin-1 expression by adding LDL cholesterol, and verified cholesterol accumulation via filipin staining (Fig. 3H and 3I). As expected, caveolin-1 expression was increased after cholesterol loading in immortalized astrocytes (Fig. 3 J and 3 K) and increased in a dose-dependent manner in baby hamster kidney (BHK) broblast cells (Supplementary Fig. 3H). Because APOE4 is associated with greater cholesterol accumulation than APOE3,[49] we examined whether caveolin-1 protein levels were higher in the cortical tissues of APOE4-TR mice than in those of APOE3-TR mice at 8 and 18 months of age (Fig. 3L). In addition, cortices from 22-month-old APOE3-TR and APOE4-TR mice were fractionated using TBSX and guanidine HCL to isolate soluble membrane proteins and insoluble aggregated proteins (Supplementary Fig.4A). Caveolin-1 protein levels in the membrane protein-enriched fraction were significantly higher in APOE4-TR mice than in APOE3-TR mice (Fig.3L). No differences in caveolin-1 levels were observed in the insoluble aggregated protein fraction (Fig. 3L). In addition, higher caveolin-1 protein levels were found in the cortex of APP/PS1/APOE4 mice than in that of APP/PS1/APOE3 mice (Fig.3M). In homogenates of human postmortem middle frontal lobe tissues from ROS, caveolin-1 protein levels were higher in APOE3/3 and APOE3/4 carriers with AD dementia compared to APOE3/3 and NCI APOE3/4 carriers in the NCI group (Fig. 3N). However, there was no difference between the APOE3/3 and APOE3/4 genotypes in either the NCI or AD group (Fig.3N). These results support that APOE4 is associated with the accumulation of cellular cholesterol in the brain asreflected by increased caveolin-1 expression.

To determine whether astrocytes were the major cell type in which ABCA1 and caveolin-1 interacted, we isolated astrocytes from the brains of adult mice. first, the myelin fraction was separated by gradient centrifugation, followed by the removal of microglia using CD11b-coupled magnetic microbeads. Astrocytes were found to be enriched in the residual fraction (Supplementary Fig. 4B). CD11b^+^ cells were enriched in Iba-1 expression, consistent with the microglial population, whereas CD11b^−^ cells were enriched in GFAP expression, consistent with the astrocyte population. Myelin fraction was enriched in beta-3 tubulin expression, consistent with enriching myelinated axons (Supplementary Fig. 4C). ABCA1 was mainly expressed in the astrocyte-enriched and axon-enriched fractions, whereas lower ABCA1 expression was detected in microglial cells (Supplementary Fig. 4D). Meanwhile, caveolin-1 was mainly expressed in astrocytes and microglia but not in axon-enriched fractions (Supplementary Fig.4E). These results confirmed that the interaction between ABCA1 and caveolin-1 is largely a feature of astrocytes.

### Lower ABCA1 function and lysosomal cholesterol accumulation induces mTORC1 activation

**Figure 4:**
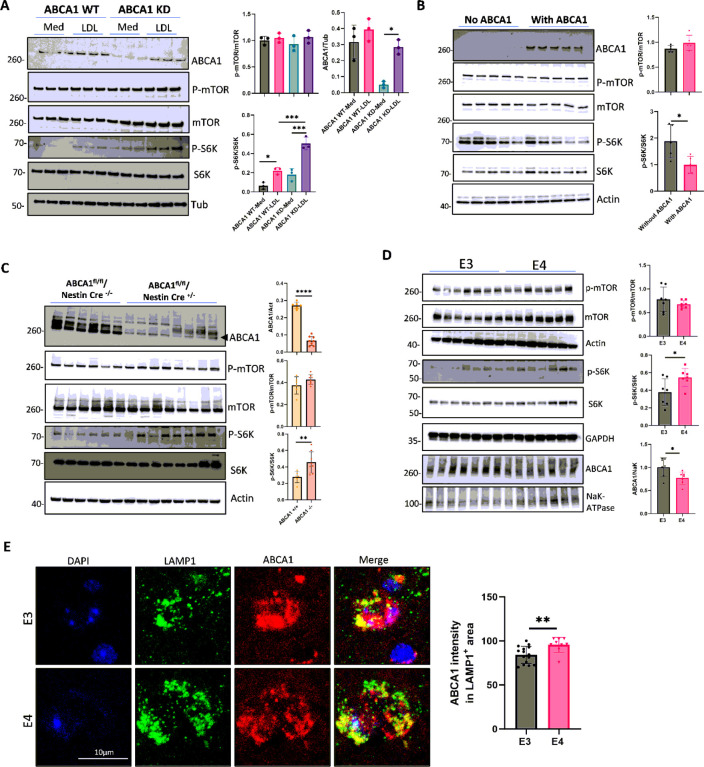
Lower ABCA1 expression increases mTORC1 activation. (**A**) ABCA1 knock down astrocytes were treated with MβCD (5 mg/mL) for 2 h, followed by loading with medium (Med) or LDL (100μg/mL) for 2 h. ABCA1, total and phosphorylated mTOR, and S6K were detected by WB (n=3 biological replicates). (**B**) BHK cells were treated with mifepristone (0.1nM) for 16 hours to induce ABCA1 expression. The cells were then treated with LDL (100μg/mL) for 24 h. ABCA1, total and phosphorylated mTOR, and S6K were detected by WB (n=5 biological replicates). (**C**) The cortex of 10-month-old ABCA1 brain conditional knockout mice was homogenized in RIPA buffer. ABCA1, total and phosphorylated mTOR, and S6K were detected by WB. (n=6 for ABCA1 WT mice, n=8 for ABCA1 knockout mice of both sexes). (**D**) ABCA1, phosphorylated and total mTOR, and S6K in the cortex of 22-months old APOE3 and APOE4-TR mice were detected by WB (n=7 for each genotype, both sexes). (**E**) Representative images of LAMP1 and ABCA1 staining by immunofluorescence in the APOE3 and APOE4-TR mouse brain slides. Scar bar, 10μm. (n=9–15 LAMP1^+^ ROIs from 3 mice in each group). Data are represented as the mean ±SD and were analyzed using a two-tailed t-test or one-way ANOVA followed by Tukey’s test. *p<0.05, ***p<0.001, ****p<0.0001.

Cholesterol accumulation in the lysosomes of a broblast cell model via ABCA1 has been shown to activate mTORC1, which plays a role in cellular senescence and SASP.[50–52] To corroborate these findings in brain cells, an ABCA1 knockdown (KD) astrocyte cell line was created using CRISPR-cas9 and cells were loaded with cholesterol. Upon cholesterol loading, the marker of mTORC1 activation p-S6K/S6K significantly increased in ABCA1 KD cells compared to WT (Fig.4A). Consistent with the ABCA1 KD cell line, overexpression of ABCA1 in BHK cells using mifepristone reduced p-S6K/S6K (Fig.4B). To validate these findings *in vivo*, the cortex of 10-month-old conditional brain ABCA1 knockout mice (ABCA1^fl/fl^ mice crossed with Nestin Cre mice) revealed significantly higher p-S6K/S6K in ABCA1 brain KO mice than in WT mice (Fig. 4C). Unlike human AD brain tissues, the brain tissues of APOE4-TR mice had lower total membrane ABCA1 expression levels than APOE3. Similar to human AD brains, APOE4-TR mice brains showed greater p-S6K/S6K (Fig. 4D), and greater colocalization of LAMP1 with ABCA1 (Fig.4E). Taken together, these findings support that cholesterol accumulation in lysosomes from either lower ABCA1 function or ABCA1 trafficking to lysosomes induces an mTORC1 activation state.

#### Reducing cholesterol rescues lysosomal ABCA1 and promotes endosome-lysosome recycling

**Figure 5:**
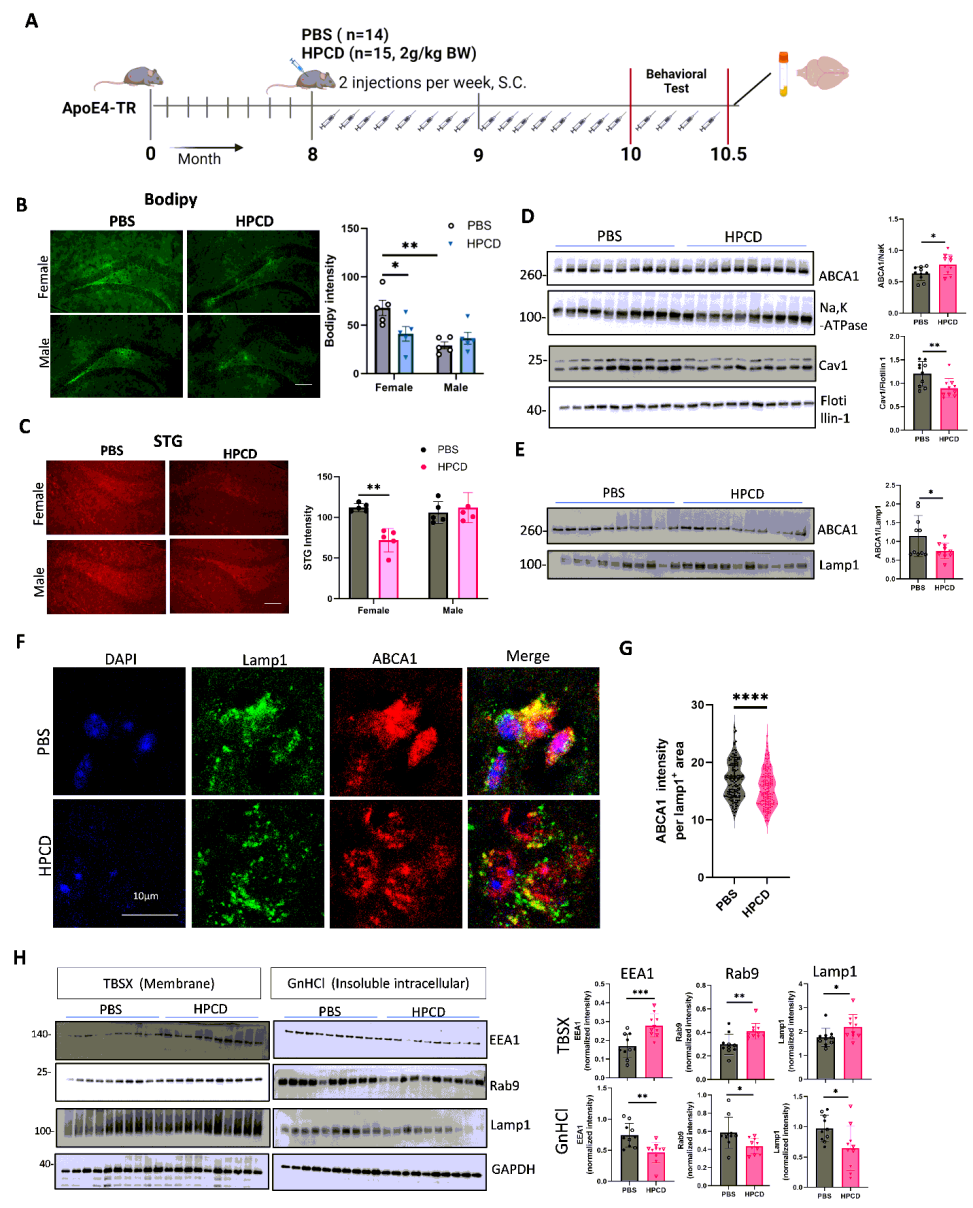
Reduction of cholesterol by cyclodextrin increases ABCA1 recycling and endolysosomal proteins in APOE4-TR mice. (**A**) Study design and time schedule of mouse experiments. APOE4-TR mice were injected with (2-Hydroxypropyl)-β-cyclodextrin (HPCD) for 2 months, followed by behavioral and pathological tests (N=14, 5 female and 9 male for PBS group; n=15, 5 females and 10 males for the HPCD group). (**B**) Cholesterol accumulation in mouse brain slides stained with BODIPY-cholesterol. Scar bar, 100μm. (**C**) Lipofuscin in mouse brain slides stained with STG. Scar bar, 100μm. (**D**) Total membrane protein levels of ABCA1 and caveolin-1 in the mouse cortex were detected by WB (n=10; five females and five males in each group). (**E**) Lysosomes were isolated from mouse brains. ABCA1 protein levels in lysosomes were detected by WB (n=10, ve females and ve males in each group). (**F**) Representative images of LAMP1 and ABCA1 staining in mouse brain. Scar bar, 10μm. (**G**) Quantification of ABCA1 intensity in LAMP1^+^ cells. (n=150 Lamp1^+^ ROI from 5 mice in each group; A.U.). (**H**) Protein levels of EEA1 (early endosome marker), Rab9 (late endosome marker), and Lamp1 (lysosome marker) in the membrane fraction (TBSX) and the intracellular fraction (GnHCl) were detected by WB. (N=10, five females and five males in each group for B, C, D, E, and H). Data are represented as mean± SD and were analyzed using an unpaired t-test. *p<0.05; ** p<0.01, *** p<0.001.

Reducing cellular cholesterol by cyclodextrin reduces caveolin-1 expression[53]. To test whether reducing cellular cholesterol rescues ABCA1 trapped in lysosomes, APOE4-TR mice were treated with PBS or 2-hydroxypropyl-β-cyclodextrin (HPCD) for 2 months starting at 8 months of age (*n = 14* PBS-and *n = 15* HPCD-treated mice, Fig. 5A). Mice body weight was not affected by HPCD treatment (Supplementary Fig. 5A). Bodipy-cholesterol staining showed that cholesterol levels in the hippocampus were lower in APOE4-TR HPCD-treated females, but not in males, compared to the PBS treatment group (Fig. 5B). Similar to bodipy-cholesterol staining, aggregated lipids stained by STG were lower in HPCD-treated females but not in males (Fig.5C). Mouse brain tissues were fractionated using TBSX and guanidine HCl to isolate the soluble membranes and insoluble aggregated proteins, respectively. Total membrane caveolin-1 levels were significantly decreased in HPCD treated-APOE4-TR mice (Fig. 5D). This was associated with enhanced ABCA1 recycling, as evidenced by the higher membrane ABCA1 levels in the HPCD treatment group than in the control group (Fig. 5D). We then isolated lysosomes from mouse brains (Supplementary Fig. 5C) and confirmed that lysosomal ABCA1 levels decreased after HPCD treatment (Fig. 5E), and further confirmed lysosomal ABCA1 sequestration by double staining for ABCA1 and Lamp1 (Fig.5F and 5G).

As increased ABCA1 activity induces small HDL formation to facilitate cholesterol efflux,[54] we measured the concentration of total HDL particles of different sizes in the cerebrospinal fluid (CSF) and plasma of HPCD-treated mice using ion mobility (Supplementary Fig.5D). The concentration of small HDL increased, while middle-sized HDL decreased in HPCD-treated mice compared to control mice in CSF (Supplementary Fig. 5E) and plasma (Supplementary Fig. 5F) although these changes did not reach statistical significance.

Since HPCD treatment reduced STG-stained aggregated proteins and lipids, we tested whether endolysosomal markers were increased in the membrane fraction compared to those in the aggregated cellular fractions. HPCD treatment resulted in more soluble and less aggregated levels of EEA1 (early endosomes), Rab9 (late endosomes), and LAMP1 (lysosomes) in the corresponding brain fractions (Fig. 5H). Together, these results indicated that the reduction of cholesterol accumulation by cyclodextrin reduced the proportion of ABCA1 trapped in aggregated lysosomes, restored endosome-lysosomal recycling, and allowed ABCA1 to recycle back to the plasma membrane and efflux cholesterol to HDL.

#### Reduction of cholesterol by cyclodextrin decreases mTORC1 activation and neuroinflammation in APOE4-TR mice

**Figure 6:**
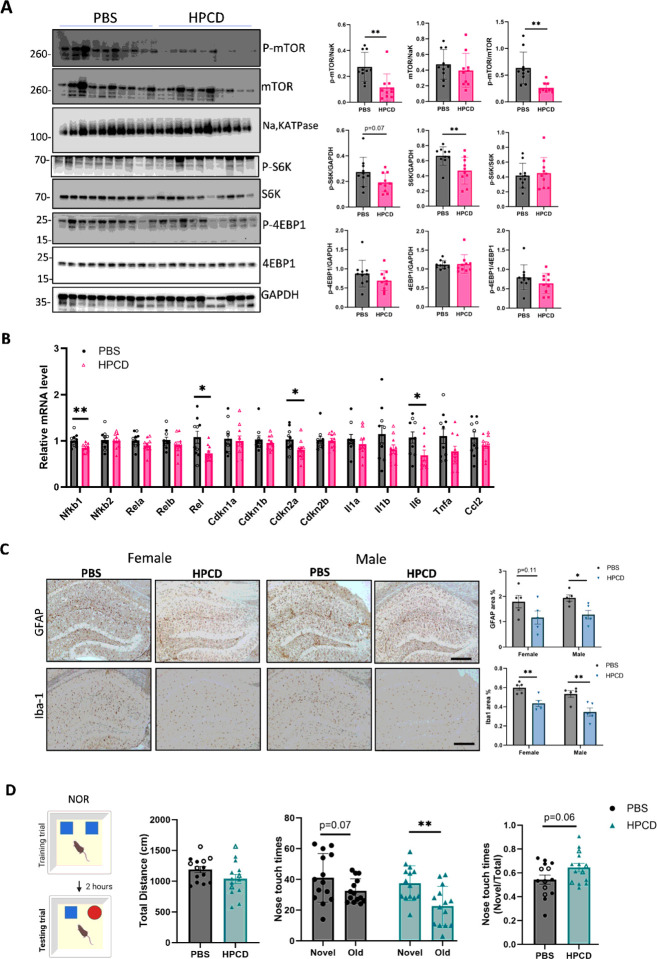
Reduction of cholesterol by cyclodextrin reduces mTORC1 activation and neuroinflammation in APOE4-TR mice. (**A**) Phosphorylated and total mTOR, S6K, and 4EBP1 in the cortex of APOE4 mice treated with or without cyclodextrin were detected by WB (n=10 for each group). (**B**) The mRNA levels of NF-κB, cellular senescence, and cytokines in the mouse cortex were determined using qPCR (n=10 for each group). (**C**) Representative slides and quantification of activated astrocytes and microglia stained with GFAP and Iba-1 antibodies, respectively, in the hippocampi of mice treated with PBS or HPCD (n=10 per group). (**D**) Recognition ability of mice treated with PBS or HPCD was tested using the novel objective recognition (NOR) test. The total distance moved during the test trial, nose touch times for both test trial objectives, and the ratio of novel to total nose touch times in the testing trial were analyzed using the EthoVision XT software(n=14 for each group).

Lowering cellular cholesterol by HPCD treatment decreased the expression of mTORC1 activation markers (Fig. 6A). As the NF-kB family is located in the central axis of the SASP [5]. NF-kB mRNA levels measured by qPCR were assessed after treatment. Three NF-kB members, Nfkb1, Rela, and Rel, were reduced in the brains of HPCD-treated mice compared to the control mice (Fig.6B). In addition, the senescence and SASP markers, such as Cdkn2a (p16) and Il-6 mRNA levels, were also decreased in the brains of HPCD-treated mice compared to the control mice (Fig.6B). In addition, activated astrocytes and microglia significantly decreased following HPCD treatment (Fig. 6C). To validate these findings in human cells, we cultured human induced pluripotent stem cell (hiPSC)-derived astrocytes with the APOE4/4 genotype (Supplementary Fig. 6A) and treated them with methyl-β-cyclodextrin (MβCD) after LDL loading. As expected, cellular cholesterol accumulation was significantly decreased following MβCD treatment (Supplementary Fig. 6B). Importantly, IL-1β and CCL2 mRNA levels were significantly reduced in the MβCD treatment group compared to those in the LDL group in hiPSC astrocytes stimulated with TNF-α and IFN-γ (Supplementary Fig. 6C). Thus, reduction in cholesterol ameliorates some of the neuroinflammatory features of senescent cells.

#### Reduction of cholesterol by cyclodextrin increases functional neuronal markers and rescues recognition memory deficit in APOE4-TR mice

Inducing ABCA1 recycling using the peptide CS-6253 in APOE4-TR mice enhanced synaptophysin and vGlut1 expression and animal behavior [55]. Similarly, we found that synaptophysin levels measured by immunofluorescence staining and western blotting were significantly increased in HPCD-treated APOE4-TR mice compared with controls (Supplementary Fig. 7A and 7B). PSD95 and vGluT1 (Vesicular glutamate transporter 1) were also significantly increased after treatment (Supplementary Fig.7B). The novel object recognition (NOR) test performed eight weeks after treatment with HPCD (Fig. 6D) showed significant differences in nose touch times between the novel and old objects, and a higher discrimination index for novel objects compared to control mice (Fig.6D). The total distance moved did not differ between the two groups of mice (Fig. 6D).

## Discussion

This is the first report to identify increased or “sequestrated” lysosomal ABCA1 expression as mechanism of cellular senescence in the AD brain. We show that ABCA1 expression strongly correlates with cellular senescence signatures of selective groups of astrocytes, excitatory neurons, microglia, and other immune cells in relation to AD neuropathology in a relatively large and well-characterized brain autopsy study. Using cellular and animal models, the accumulation of cellular cholesterol and sequestration of ABCA1 in lysosomes led to an increase in cellular senescence markers and SASP via mTORC1 activation.

Whether cellular senescence is a cause or consequence of neurodegeneration remains unclear [2], but growing evidence confirms the presence of cellular senescence in different cell types, including neurons, astrocytes, microglia, endothelial cells, and oligodendrocyte precursor cells in AD mouse models and, importantly, in AD human brains [4, 10, 56–59]. Our analysis is consistent with another study using single-cell transcriptomic data, which showed that excitatory neurons are the most senescent cells in the human brain [60]. Aβ and Tau accumulation are major factors that induce cellular senescence in AD [8, 59]. Removing senescent cells genetically or pharmacologically ameliorates AD pathology and improves memory in AD mouse models [9, 10]. Whether cells that do not display senescent phenotypes, such as inhibitory neurons, are more vulnerable to cell death remains unknown.

Dysfunction in cholesterol homeostasis drives cellular senescence in AD. A high-fat diet increases senescent endothelial cells in LDLR (low-density lipoprotein receptor) knockout mice [61] and upregulates SASP in the kidneys of C57BL/6J mice [62]. 27-hydroxycholesterol accelerated cellular senescence in resident human lung cells in an in vitro assay [18]. Cholesterol and lipid accumulation has been reported in cellular and mouse models of Aβ [63, 64], tau [65, 66] and APOE4 [39, 67–69]. A recent study identified more senescent neurons in the hippocampus of old ApoE4-TR mice than in ApoE3-TR mice owing to dysregulation of ATP and acetyl coenzyme A [14], which are substrates for cholesterol synthesis [70]. Cholesterol localization shifts to lysosomes, leading to the formation of microdomains enriched for the mTORC1 scaffolding complex, priming mTORC1 for activation, and increasing proinflammatory SASP [5, 71]. ABCA1 plays a pivotal role in importing cholesterol into lysosomes, thereby causing lysosomal dysfunction [71]. ABCA1 expression in lysosomal compartments is implicated in the accumulation of lipid droplets in astrocytes and microglia [72]. ABCA1 degradation is mediated by caveolin-1, the principal protein marker for caveolae, which regulates high-density lipoprotein (HDL) metabolism and cholesterol efflux [73]. Importantly, caveolin-1 can act as a cholesterol sensor and regulate cellular cholesterol homeostasis [73, 74] and a potential biomarker for effective reducing sequestrated brain cholesterol pools.

Our results suggest that restoring cholesterol homeostasis is a potential therapeutic strategy for AD. A lower cholesterol burden induced by cyclodextrin increases axonal myelination and improves learning and memory in APOE4 mice [69]. However, cyclodextrin is not a potent cholesterol chelator and toxic when used at higher doses. In AD mouse models, the reduction of cholesterol in astrocytes robustly reduces amyloid and tau burden [75]. Clearing out of the cholesterol burden promotes the clearance of defective mitochondria via autophagy in cells and ameliorates brain inflammation and neurodegeneration in the APOE4/Tau mouse model [65, 76].

Our findings suggest that regulation of cholesterol metabolism, particular cholesterol efflux appears to be a druggable target for AD. Unfortunately, no clinical interventions focusing on modulating cholesterol metabolism have been successful in APOE4 carriers with AD. Examples of these interventions include rosiglitazone [77], pioglitazone (PPARγ agonists) [78], and bexarotene (RXR agonist) [79], which are ineffective for APOE4 carriers with AD. The challenge with such interventions is that drugs that induce both ABCA1 and APOE expression without accounting for endolysosomal dysfunction may inadvertently accelerate ABCA1 lysosomal sequestration in APOE4 or in conditions of impaired lysosomal recycling.

Our study has a few limitations. We do not identify the mechanisms by which APOE4 induced cholesterol accumulation affects lysosomal recycling. Prior studies implicate that targeting NHE6, the primary proton leak channel in the early endosome can reverse the ApoE4-induced recycling block of several receptors[80]. It is plausible that cholesterol directly or indirectly affects endolysosomal pH and recycling. With the relatively small sample size of CSF obtained from mice studies, the changes in HDL particles after treatment did not reach statistical significance. Finally, caveolin-1 expression does not appear to be a good drug target to restore ABCA1 recycling, as reducing its expression was associated with lower cholesterol efflux.

## Conclusions

**Figure 7:**
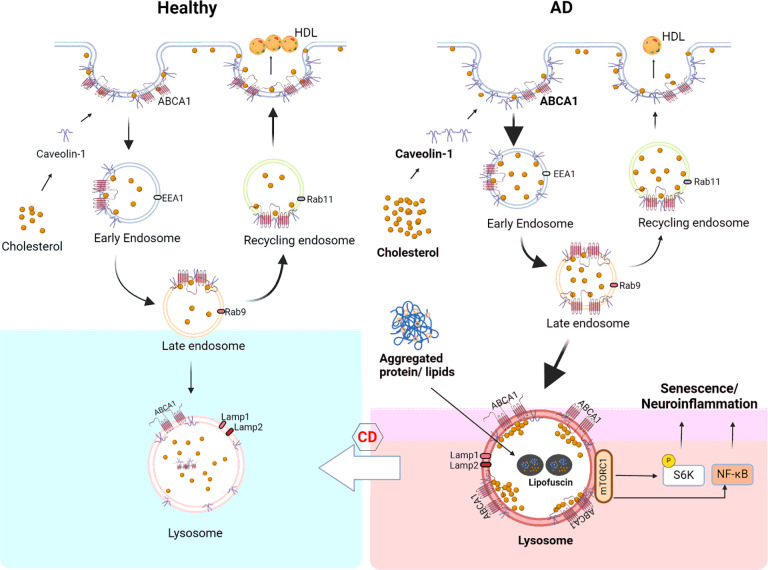
Cholesterol accumulation promotes senescence and neuroinflammation through lysosomal ABCA1 aggregation in APOE4 and AD cells. Compared to healthy individuals, a higher cholesterol burden in AD (including APOE4) induces more caveolin-1 expression, traps more ABCA1 in endosomes and lysosomes, decreases plasma membrane ABCA1 levels, slows down endosome-lysosome recycling, and promotes cholesterol and lipid accumulation in lysosomes, leading to senescence features and neuroinflammation through mTORC1 activation. Reduction of cholesterol by cyclodextrin reduces lysosomal ABCA1 levels and increases ABCA1 recycling to the plasma membrane to promote the efflux of cholesterol. Lowering the cholesterol burden in lysosomes reduces mTORC1 activation to decrease senescence and neuroinflammation. Illumination is created by BioRender.

Cholesterol accumulation in cells is normally compensated for by enhancing ABCA1 recycling into the plasma membrane, allowing the efflux of cholesterol to lipoproteins for exchange among different cell types and cholesterol clearance. Here, we demonstrate that cholesterol accumulation in APOE4 and AD induces greater expression of caveolin-1, which endocytoses and traps ABCA1 in lysosomes, activating the mTORC1 pathway and inducing cellular senescence. Reducing cholesterol by cyclodextrin decreased lysosomal ABCA1, accelerated endolysosomal recycling, increased the efflux of cholesterol to HDL, and reduced mTORC1 activation and senescence-associated neuroinflammation (Fig.7). These findings highlight the importance of drugs or therapeutics that target lysosomal functions and ABCA1 recycling to attenuate cellular senescence in the brain.

## Data Availability

Data are available from the corresponding author upon request. Analytical codes can be accessed by emailing the corresponding author. Sharing data from ROSMAP will follow ROSMAP data-sharing policies.

## Abbreviations

AD: Alzheimer disease
APOE: Apolipoprotein E
ROSMAP: Religious order study/memory aging project
SASP: Senescence-associated secretory phenotype
ABCA1: ATP-binding cassette A1
sn-RNA seq: single-nucleus RNA sequencing
iPSC: induced pluripotent stem cells
APOE-TR: APOE-target replacement
DLPFC: Dorsolateral prefrontal cortex
NCI: No cognitive impairment
MCI: Mild cognitive impairment
ssGSEA: single-sample Gene Set Enrichment Analysis
CERAD: Consortium to Establish a Registry for Alzheimer’s Disease
mTOR: mammalian target of rapamycin
mTORC1: mTOR complex 1
NFT: Neurofibrillary tangles
STG: SenTraGor
GFAP: Glia brillar acid protein
rE3/rE4: recombinant ApoE3/ApoE4
GFP: Green uorescent protein
LC-MS/MS: Liquid chromatography–mass spectrometry
SDS-PAGE: Sodium dodecyl-sulfate polyacrylamide gel electrophoresis
KEGG: Kyoto Encyclopedia of Genes and Genomes
DAVID: Database for Annotation, Visualization, and Integrated Discovery
Cav-1: Caveolin-1
AP2B1: Adaptor-related protein complex 2 subunit beta 1
BHK: Baby hamster kidney
HPCD: 2-hydroxypropyl-β-cyclodextrin
HDL: High-density lipoproteins
EEA1: Early endosome antigen 1
LAMP1: Lysosomal-associated membrane protein 1
NF-kB: Nuclear factor kappa-light-chain-enhancer of activated B cells
MβCD: methyl-β-cyclodextrin
LDL: Low-density lipoproteins
TNF-α: Tumor necrosis factor alpha
IFN-γ: Interferon gamma
vGlut1: Type 1 vesicle glutamate transporter
PSD95: Postsynaptic density protein 95
NOR: Novel object recognition
A.U.: arbitrary unit
WB: western blotting
ROI: Region of interest
